# Hypermethylation of miR-205-5p by IR Governs Aggressiveness and Metastasis via Regulating Bcl-w and Src

**DOI:** 10.1016/j.omtn.2018.12.013

**Published:** 2018-12-31

**Authors:** Eun Sook Kim, Jae Yeon Choi, Su Jin Hwang, In Hwa Bae

**Affiliations:** 1 Division of Radiation Biomedical Research, Korea Institute of Radiological & Medical Sciences, Seoul 01812, Republic of Korea

**Keywords:** radioresistance, Bcl-w, miR-205-5p, hypermethylation, metastasis

## Abstract

Although radiotherapy has been successfully applied to treat many cancer types, surviving cancer cells often acquire therapeutic resistance, leading to increased risk of local recurrence and distant metastases via modification of the tumor microenvironment. Previously, we reported that high expression of Bcl-w in cancer patients is significantly correlated with poor survival as well as malignant activity. However, the relationship between ionizing radiation (IR)-induced resistance and Bcl-w expression in cancer cells is currently unclear. We showed that IR-induced Bcl-w contributes to EMT (epithelial-mesenchymal transition), migration, angiogenesis, stemness maintenance, and metastasis by promoting the expression of factors related to these phenotypes, both *in vitro* and *in vivo*. Meanwhile, IR enhanced hypermethylation of miR-205-5p CpG islands through Src activation, leading to decreased miR-205-5p expression and, in turn, potentially stimulating Bcl-w-mediated malignant activity and metastasis. The clinical applicability of Bcl-w and miR-205-5p from cells or animal models was confirmed using tissues and plasma of breast carcinoma patients. Based on the collective findings, we propose that miR-205-5ps as important negative mediators of resistance in radiotherapy could serve as useful potential targets of concurrently applied genetic therapy aimed to inhibit tumor aggressiveness and enhance the efficiency of radiotherapy in cancer patients.

## Introduction

Ionizing radiation (IR) is widely used as a therapeutic option for many cancer types, but surviving cancer cells often acquire resistance after radiotherapy.^1^ Subsequently, the tumor microenvironment is modified, affecting multi-cellular responses, tissue remodeling, local recurrence, and distant metastases.^1^, ^2^, ^3^, ^4^, ^5^, ^6^, ^7^, ^8^, ^9^ However, IR stimulates tumor progression in a number of cancer types, including leukemia, lymphoma, sarcomas, thyroid, skin, lung, and breast cancers,^10^, ^11^ ultimately leading to therapeutic failure.

Bcl-w (B cell lymphoma-w) is upregulated in gastric, colorectal, breast, cervical, lung, and bladder cancers, and glioblastoma multiforme^12^, ^13^, ^14^, ^15^, ^16^, ^17^, ^18^, ^19^, ^20^ and acts as a potential mediator of resistance to several chemotherapeutic drugs owing to its activity in preventing cell death^21^ as a pro-survival factor. Bcl-w promotes tumor progression and stemness in gastric cancer cells and glioblastoma multiforme^15^, ^17^, ^22^ as a pro-oncogene.^23^ However, the relationship between IR-induced resistance and Bcl-w expression in cancers is currently unclear.

MicroRNAs (miRNAs) regulate gene expression at the post-transcriptional level by binding the 3′ UTR of specific target mRNAs.^24^, ^25^, ^26^ These small non-coding RNA molecules are involved in several biologic and pathologic processes, such as development, proliferation, differentiation, and apoptosis.^27^ Accumulating evidence shows that miRNAs can act as either oncogenes^28^, ^29^ or tumor suppressor genes,^30^ and their aberrant expression occurs frequently in various tumor types.^31^ Additionally, recent reports indicate that the expression levels of some miRNAs are significantly changed after irradiation.^32^, ^33^

To establish the effects of radioresistance on tumor progression and metastasis, we investigated whether IR stimulates Bcl-w expression and the mechanisms by which IR-induced Bcl-w promotes aggressive properties in cancer *in vitro*, *in vivo*, and in patient samples. Our collective data suggest that combining radiotherapy with genetic therapy to inhibit Bcl-w, which functions as an important mediator of resistance in radiotherapy, may present a promising strategy for enhancement of the efficiency of radiotherapy in cancer patients.

## Results

### IR Induces Bcl-w Expression in Human Cancer Cells

**Figure 1:**
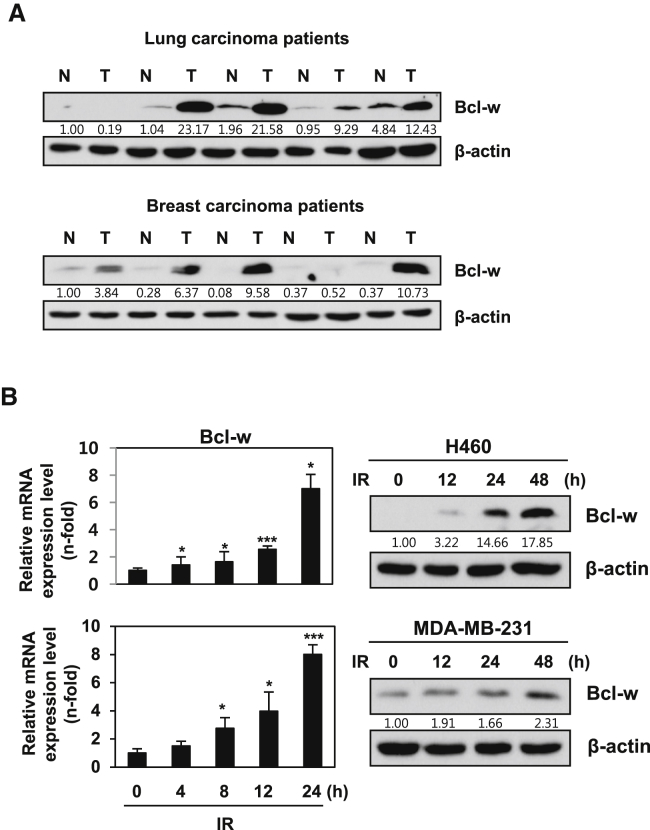
IR Increases Expression of Bcl-w, Which Is Highly Upregulated in Lung and Breast Cancer Patients (A) Western blot analysis showing the expression of Bcl-w protein in adjacent tissues (N) and cancerous tissues (T) of lung and breast cancer patients, respectively. (B) IR (5 Gy)-induced expression levels of Bcl-w mRNA (left) and protein (right) time dependently by qRT-PCR or western blot analysis, respectively, in H460 and MDA-MB-231 cells. GAPDH and β-actin always served as a loading control in qRT-PCR or western blot analysis of these results, respectively. The data are presented as the mean SD of three experiments (*p < 0.05, **p < 0.005, ***p < 0.0005, Student’s t test).

Bcl-w protein was significantly upregulated in human lung and breast cancer tissues (Figure 1A). Our data support previous findings of high Bcl-w expression in various human cancers.^12^, ^13^, ^14^, ^15^, ^16^, ^17^, ^18^, ^19^, ^20^ IR (5 Gy) augmented the mRNA and protein levels of Bcl-w in human lung (H460) and breast cancer (MDA-MB-231) cell lines in a time-dependent manner (Figure 1B). Bcl-w-depleted cells decreased IR-enhanced colony-forming ability, as determined from clonogenicity assays (Figure S1). These results support the hypothesis that IR-induced Bcl-w is linked to treatment resistance and low mortality of patients through promoting cancer cell survival.

### IR-Induced Bcl-w Promotes Mesenchymal Traits, Migratory and Angiogenic Potentials, and Stemness Maintenance

**Figure 2:**
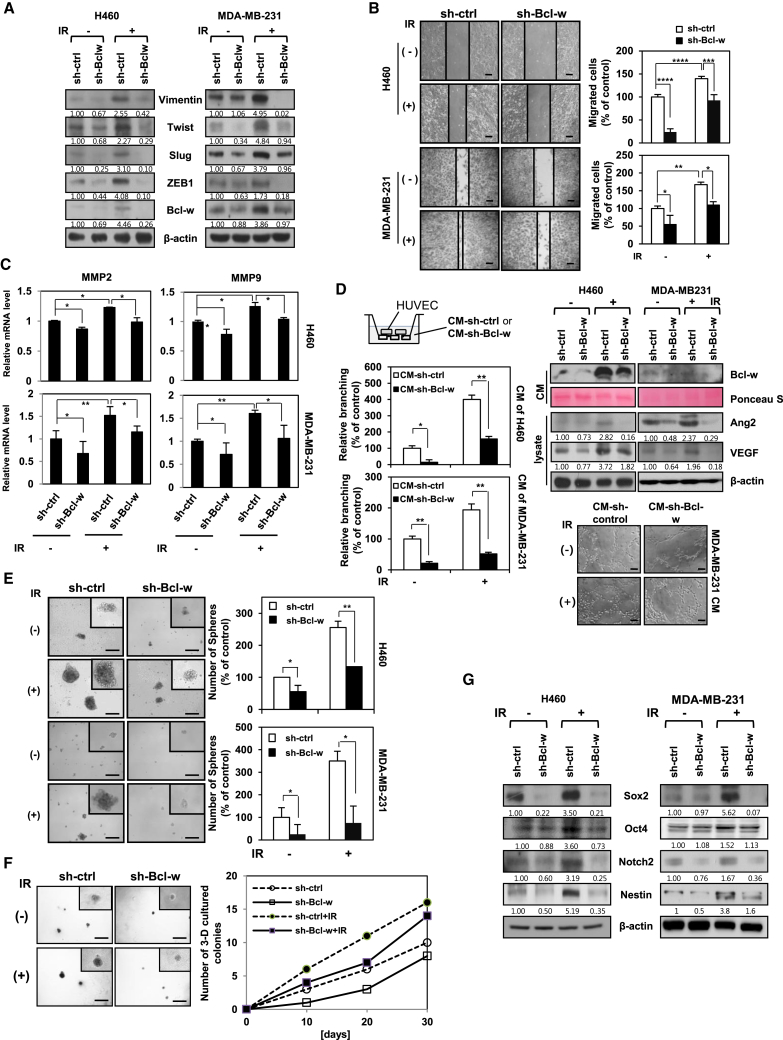
Bcl-w Is Involved in IR-Induced EMT, Migratory Potential, Angiogenesis, and the Maintenance of Stemness H460 and MDA-MB-231 cells were transfected with sh-Bcl-w or treated with IR (5 Gy). (A) Mesenchymal marker proteins containing Vimentin, Twist, Slug, and ZEB1 were determined by western blot analysis. β-actin was used as loading control. (B) After indicated cells were grown to monolayers in 6-well plates, cells were scratched and then incubated for 18 h. The confluent cells in three fields from the scratched area (200 × 500 μm^2^) were stained and counted. These experiments were performed in triplicate, and the mean value is shown in the graph (scale bar, 100 μm). (C) Levels of MMP-2 and MMP-9 mRNA were verified by q-RT PCR. (D) Left, after HUVEC treatment with conditioned media (CM) of H460 and MDA-MB-231 cells (top left), which were transfected with sh-Bcl-w or exposed with IR (5 Gy), prepared cells were seeded on Matrigel-coated 96-well plates (1 × 10^4^ cells/well) and then incubated for 6 h to assess tube-formation assay (scale bar, 100 μm, bottom right). Averaged numbers of capillary tube branches in eight random fields are counted and shown as a graph. Top right, levels of Bcl-w secreted to conditioned media were measured. Ponceau S was used as loading control. Angiogenesis-related proteins Ang2 and VEGF were detected by western blot analysis. β-actin was used as loading control. (E) Indicated treated cells were seeded onto 100-mm culture dishes (1 × 10^3^ cells/dish) and cultured for 5∼10 days. These experiments were performed in triplicate, and the mean value was shown (scale bar, 500 μm). (F) Indicated cells were seeded on a pre-coated 24-well plate with 60 μL of Matrigel and grown for 30 days in H460 cells. Colony number was counted every 10 days. These experiments were performed in triplicate (scale bar, 500 μm). (G) Cancer stem-like cell marker proteins containing Sox2, Oct4, and Notch2 were determined in H460 and MDA-MB-231 cells by western blot analysis. β-actin was used as loading control. All data are expressed as the mean ± SD of three experiments. (*p < 0.05, **p < 0.005, ***p < 0.0005, ****p < 0.00005, Student’s t test).

To determine whether Bcl-w contributes to the acquisition of IR-induced aggressive properties in cancer cells, we evaluated its effects on IR-mediated tumorigenicity, including mesenchymal-related traits, migration, angiogenesis, and maintenance of stemness. Knockdown of Bcl-w using small hairpin RNA (shRNA) in H460 and MDA-MB-231 cells suppressed IR-induced expression of the mesenchymal marker proteins (Figure 2A) and exhibited a decrease in the IR-induced migratory potential and invasive properties (Figure 2B) through downregulation of the mRNA levels of the matrix metalloproteinases (MMPs) MMP-2 and MMP-9, which were associated with tumor invasiveness (Figure 2C).^34^ To evaluate the effect of IR-induced Bcl-w on angiogenesis, we performed the tube formation assay *in vitro*, which is widely used as a model for the reorganization stage of angiogenesis.^35^ For the assay, we collected conditioned medium (CM) from H460 and MDA-MB-231 cells transfected with sh-Bcl-w or treated with IR (Figure 2D, left) and detected the level of Bcl-w secreted in each CM (Figure 2D, right). Secreted Bcl-w in CM from Bcl-w-depleted cells treated with IR was lower than that in CM from IR-treated cells. Human umbilical vein endothelial cells (HUVECs) suspended in CM of Bcl-w-depleted cells displayed attenuated tube formation ability, compared to those in CM from IR-treated cells (Figure 2D, left). The tube formation ability of HUVECs was positively correlated with the secreted Bcl-w level. Expression the angiogenesis-related proteins, angiopoietin-2 (Ang-2) and vascular endothelial growth factor (VEGF), was downregulated in Bcl-w knockdown cells exposed to IR, relative to IR-only-treated cells (Figure 2D, right). Our findings indicate that Bcl-w is involved in IR-mediated acquisition of angiogenic properties in human cancer cells. To investigate the role of IR-induced Bcl-w in maintenance of stemness, two cells were transfected with sh-Bcl-w or treated with IR, and growth and expression of cancer stem-like cell markers were measured using sphere-forming and three-dimensional culture assays and western blotting. Notably, the ability of sphere or colony formation was decreased in sh-Bcl-w knockdown cells treated with IR, compared to IR-only-treated cells (Figures 2E and 2F). Moreover, expressions of cancer stem-like cell markers were attenuated in Bcl-w-knockdown cells (Figure 2G).^36^, ^37^ Based on the data, we suggest that Bcl-w mediates IR-induced maintenance of stemness in a stem-like cell population of human cancer cells.

### IR-Induced Bcl-w Promotes Primary Breast Tumor Progression and Pulmonary Metastasis in Nude Mice

**Figure 3:**
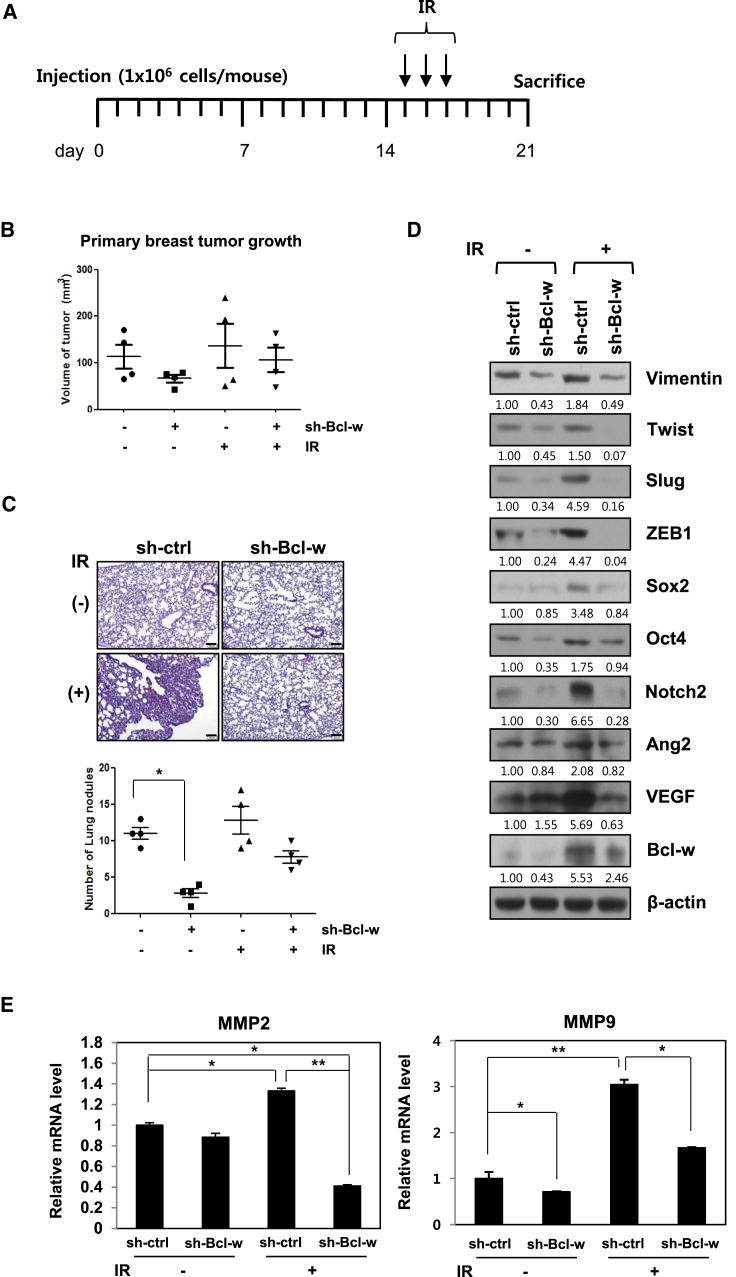
IR-Induced Bcl-w Promotes Primary Breast Tumor Formation and Pulmonary Metastasis in Nude Mice (A) The plan of animal experiments was described, in which sh-Bcl-w-transfected MDA-MB-231 cells (1 × 10^6^ cells/mouse) were orthotopically injected into mammary fat pads of 8-week-old nude mice. After implantation, mice were exposed to IR three times per 2.5 Gy and then sacrificed at 21 days. (B and C) Primary breast tumor growth (B) and pulmonary metastatic nodules (scale bar, 100 μm) (C) are shown as box-and-whisker plots, and pulmonary tissues of mice were stained with H&E (C, top) (n = 4 animals per group), respectively. (D and E) Using metastatic pulmonary tissues of mice, IR-induced Bcl-w-regulated signaling components were detected by western blot analysis (D) or qRT-PCR (E), respectively. The data are presented as the mean SD (*p < 0.05, **p < 0.005, ***p < 0.0005, Student’s t test).

To determine the effects of IR-upregulated Bcl-w on metastasis *in vivo*, Bcl-w knockdown or negative-control metastatic MDA-MB-231 cells were implanted orthotopically into nude mice followed by exposure to IR (2.5 Gy) on days 15, 16, and 17. Mice were sacrificed 21 days after implantation (Figure 3A). Fractionated radiation treatment offers a significant advantage owing to fewer toxic effects on normal tissue^38^ and was therefore selected to avoid exposing mice to the acute dose. A previous study by our group showed that the biological events in cells exposed to a single dose (5 Gy) were similar to those in cells receiving fractionated doses (2.5 Gy × 3 times).^39^ The size of primary breast tumors and number of metastatic nodules in the lung decreased dramatically in mice injected with Bcl-w knockdown cells, compared with those administered fractionated IR-treated or wild-type cells (negative control mice) (Figures 3B and 3C). Histological examination of metastatic pulmonary tissues of mice using H&E staining revealed lower density in mice injected with sh-Bcl-w-transfected cells, compared to those with IR-treated or negative control cells (Figure 3C, left). In addition, mesenchymal markers, angiogenesis-related proteins, and cancer stem-like cell marker proteins and MMP-2/-9 mRNAs were upregulated in metastatic pulmonary tissues treated with IR (Figures 3D and 3E). Immunohistochemical analysis showed that IR-induced Bcl-w promotes the expression of major components of metastasis, including Slug, MMP-9, Sox2, and Ki-67 (Figure S2). Our data provide compelling evidence that IR-induced Bcl-w enhances primary breast cancer metastasis to lung by stimulating the expression of components related to mesenchymal traits, migration, angiogenesis, stemness maintenance, and proliferation and mediates the metastatic capacity of cancer cells *in vivo*.

### Bcl-w Is a Direct Target of miR-205-5p, Which Is Downregulated by IR

**Figure 4:**
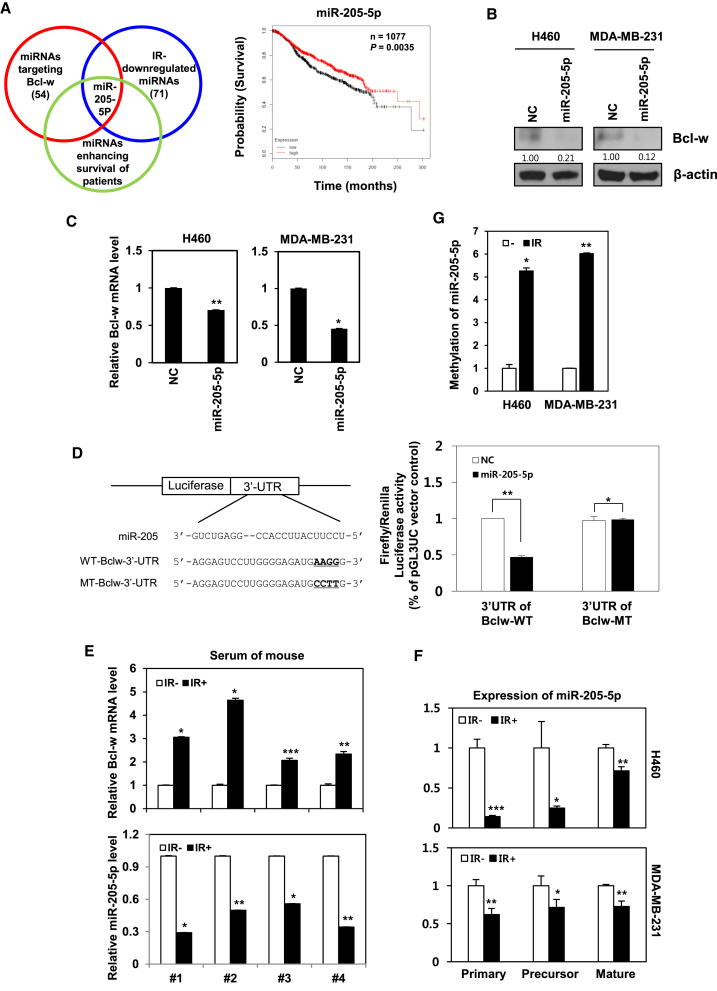
Bcl-w Is a Direct Target Gene of miR-205-5p, Which Is Downregulated by IR (A) Left, Venn diagram illustrating selected miR-205-5p, which is downregulated by IR and enhances the survival of patients as well as targets Bcl-w. Right, the probability of patient survival for miR-205-5p, which fulfilled above conditions, was searched using Kaplan-Meier analysis. Kaplan-Meier survival curves of miR-205-5p expression in breast cancer patients were based on METABRIC dataset. (B and C) Validation by western blot analysis and qRT-PCR of Bcl-w protein (B) and mRNA (C) expression after transfection of miR-205-5p in H460 and MDA-MB-231 cells. (D) Left, structure of reporter constructs containing Bcl-w 3′ UTR downstream of the luciferase ORF. Right, luciferase assay of MDA-MB-231 cells, transfected with firefly luciferase constructs containing wild-type (WT) or mutant type (MT) target sites of Bcl-w, and negative control or miR-205-5p are shown. (E) After metastatic MDA-MB-231 cells (1 × 10^6^ cells/mouse) were injected into 8-week-old nude mice, IR (three times per 2.5 Gy) was exposed to mice, and then blood of mice was collected at 21 days. Levels of Bcl-w mRNA and miR-205-5p expression were measured in serum of mice by qRT-PCR (n = 4 animals per group). miRNA level was normalized with U6, and mRNA level was normalized with GAPDH. (F) After IR exposure, the biogenesis of miR-205-5p was examined by qRT-PCR in H460 and MDA-MB-231 cells. (G) Methylation-specific PCR (MSP) for methylation status of miR-205-5p CpG islands was conducted in H460 and MDA-MB-231 cells. All data are expressed as the mean ± SD of three experiments (*p < 0.05, **p < 0.005, ***p < 0.0005, Student’s t test).

MiR-205-5p was initially identified using *in silico* miRNA target-prediction software, such as Targetscan and miRanda.^32^, ^33^ We used the Kaplan-Meier analysis^40^ to analyze the effect of mir-205-5p on patients’ overall survival (Figure 4A). Three characteristics have been documented in relation to this miRNA, specifically, downregulation by IR, enhancement of patient survival, and suppression of Bcl-w. Inhibition of Bcl-w protein and mRNA levels by miR-205-5p was additionally verified via western blot and qRT-PCR analyses, respectively, in H460 and MDA-MB-231 cells (Figures 4B and 4C). To ascertain whether Bcl-w is a direct target of miR-205-5p, we constructed 3′ UTR reporter plasmids containing full-length Bcl-w 3′ UTR with the wild-type (WT) or mutant type (MT) miR-205-5p binding site (Figure 4D, top). miR-205-5p suppressed expression of the reporter gene containing the 3′ UTR of Bcl-w-WT but had no effect on the gene containing the 3′ UTR of Bcl-w-MT, as determined using the luciferase assay (Figure 4D, bottom). To determine the regulation patterns of Bcl-w and miR-205-5p *in vivo*, mice were injected with metastatic MDA-MB-231 cells and either left untreated (negative control) or treated with IR (three times per 2.5 Gy), following which blood was collected after 21 days. miRNAs are known to circulate in liquid biopsies containing blood.^41^, ^42^ Utilizing this feature, we determined expression levels of Bcl-w mRNA and miR-205-5p in mouse blood by qRT-PCR. Bcl-w mRNA expression was increased, while that of miR-205-5p was decreased in sera of IR-treated compared with negative control mice (Figure 4E). Our results indicate that expression of Bcl-w and miR-205-5p are negatively correlated in the animal model, consistent with the data from cancer cells *in vitro*, supporting the feasibility of clinical application for therapy. To determine the mechanism underlying downregulation of miR-205-5p by IR, its biogenesis was examined. IR dramatically suppressed both the precursor and mature forms of miR-205-5p in H460 and MDA-MB-231 cells (Figure 4F), supporting the theory that expression of Bcl-w is enhanced as a consequence of IR-induced inhibition of miR-205-5p. To determine the underlying cause of miR-205-5p suppression by IR, methylation-specific PCR (MSP), which determines the methylation status of miR-205-5p CpG islands was conducted in H460 and MDA-MB-231 cells. IR increased dramatically the hypermethylation of miR-205-5p (Figure 4G). We confirmed that IR suppressed expression of miR-205-5p by inducing hypermethylation of its CpG islands using 5-Aza-2′-deoxycytidine (5-AzadC, DNMT1 inhibitor) (Figure S3).

### miR-205-5p Attenuates IR-Induced Malignant Activity and Pulmonary Metastasis *In Vitro* and *In Vivo*

**Figure 5:**
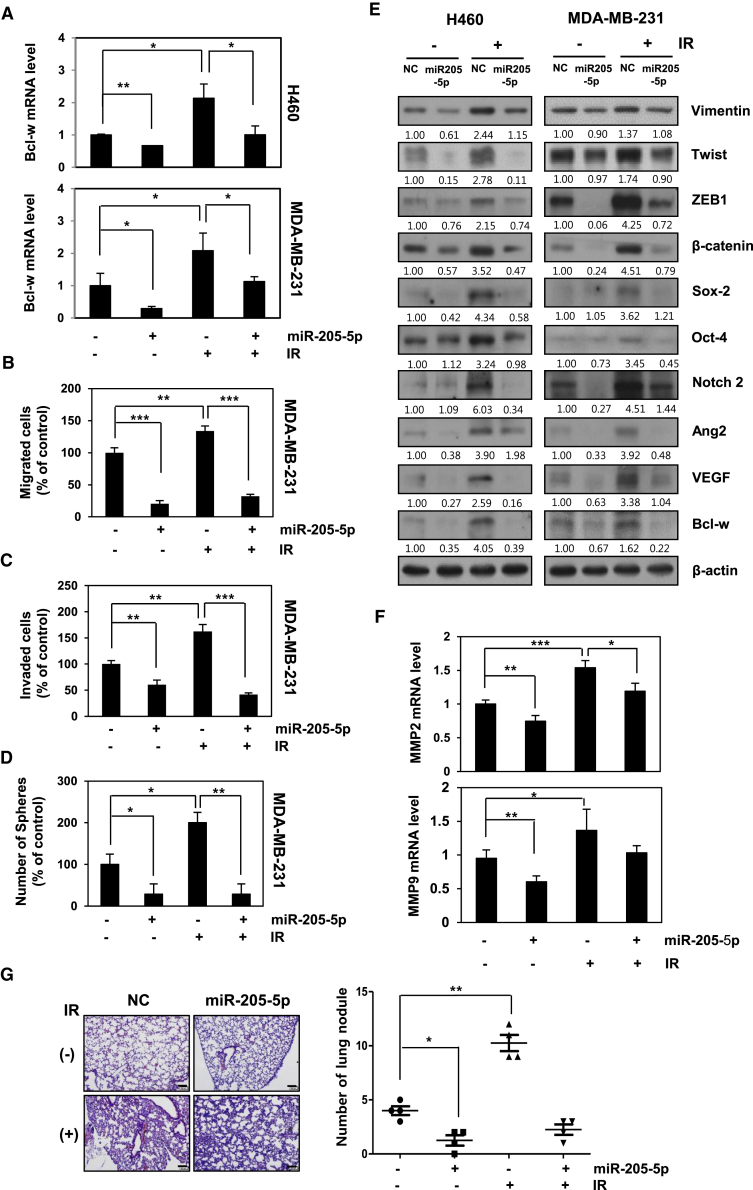
miR-205-5p Inhibits IR-Induced Tumorigenicity and Metastasis *In Vitro* and *In Vivo* (A–D) After H460 and MDA-MB-231 cells were transfected with miR-205-5p mimic or treated with IR, expressions of Bcl-w mRNA were determined using qRT-PCR (A), migration (B), invasion (C), and sphere-formation assays (D) in indicated cells. (E and F) Expressions of miR-205-5p/Bcl-w-related signaling factors by IR were determined using western blot analysis (E) and qRT-PCR (F), respectively. β-actin was used as loading control. (G) MDA-MB-231 cells transfected with negative control (NC) or miR-205-5p were transplanted into mammary fat pad of nude mice (scale bar, 100 μm. A graphical representation of the number of lung nodules is shown. All data are presented as the mean SD (*p < 0.05, **p < 0.005, ***p < 0.0005, Student’s t test).

Bcl-w mRNA levels after treatment with IR in the presence or absence of synthetic miR-205-5p were measured. The miR-205-5p mimic suppressed IR-induced Bcl-w mRNA expression in H460 and MDA-MB-231 cells (Figure 5A). To determine the role of miR-205-5p in IR-induced Bcl-w-expressing cells, we performed migration, invasion, and sphere-formation assays followed by expression analysis of signaling-related factors after treatment with IR in the presence or absence of miR-205-5p mimic. Notably, miR-205-5p-transfected cells displayed decreased migration, invasion, sphere formation, and signaling-related protein expression, compared with negative control cells. IR-treated cells containing miR-205-5p displayed decreased migration, invasion, and sphere formation as well as lower expression of signaling-related components compared with IR-only-treated cells (Figures 5B–5F). The results indicate that miR-205-5p attenuates IR-induced tumor progression through suppressing Bcl-w expression and consequent activity. Next, miR-205-5p-transfected MDA-MB-231 cells were orthotopically injected into the mammary fat pad of nude mice treated with or without IR. Based on H&E staining and graphical presentation of the lung nodule number, decreased pulmonary metastasis was detected in IR-treated mice in the presence of miR-205-5p compared to IR-only-exposed mice (Figure 5G). In view of these findings, we propose that miR-205-5p may be effectively used as a target for genetic therapy aimed at inhibiting IR-induced resistance events, such as malignant activity and metastasis.

### Clinical Application in Tissues or Plasma of Breast Cancer Patients

**Figure 6:**
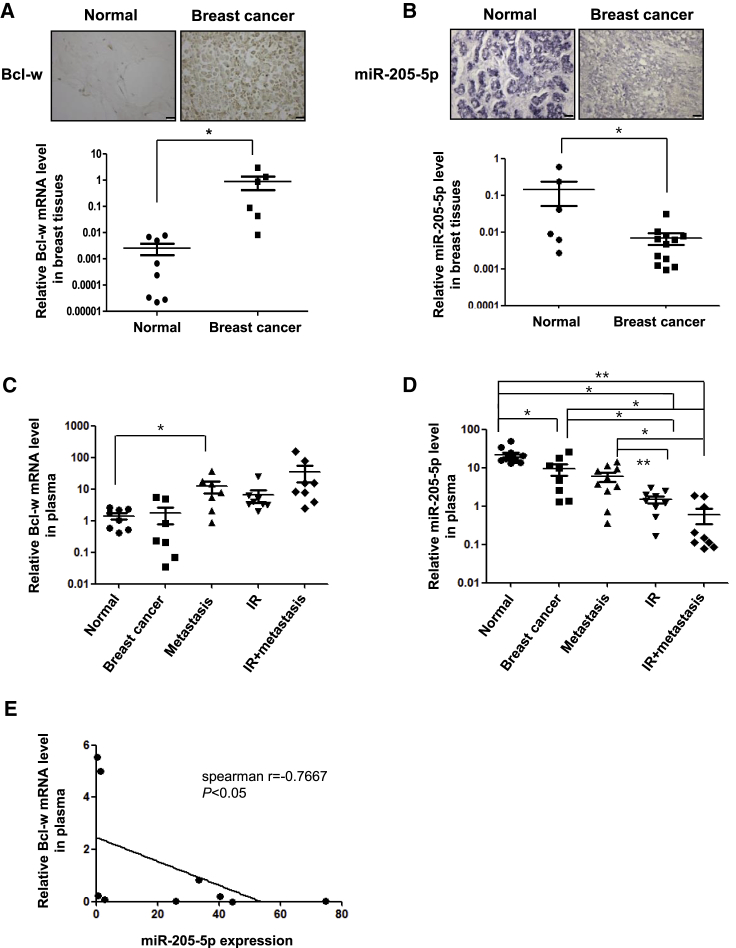
Verification of Clinical Application for This Study Using Tissues and Plasma of Breast Carcinoma Patients (A) Analysis of Bcl-w expression in eight breast cancer tissues compared to adjacent tissues by immunohistochemistry (scale bar, 100 μm) and qRT-PCR, respectively. (B) Analysis of miR-205-5p expression in 12 breast cancer tissues compared to adjacent tissues by *in situ* hybridization (purple staining) and qRT-PCR, respectively. (C and D) Using plasma of breast cancer patients in presence or absence of metastasis or with or without IR treatment, levels of Bcl-w mRNA (C) and miR-205-5p (D) expression were determined by qRT-PCR. (C) Expression of Bcl-w mRNA in normal tissues (n = 8), cancer tissues (n = 7), metastatic tissues (n = 7), IR-treated tissues (n = 8), and IR + metastatic tissues (n = 8). (D) Expression of miR-205-5p in normal tissues (n = 11), cancer tissues (n = 8), metastatic tissues (n = 10), IR-treated tissues (n = 9), and IR + metastatic tissues (n = 9). (E) Spearman correlation between the Bcl-w expression and miR-205-5p in plasma of breast cancer patients (n = 9). The Spearman correlation coefficient (r) was indicated. All data are presented as the mean SD (*p < 0.05, **p < 0.005, ***p < 0.0005, Student’s t test).

Expression of Bcl-w was increased in each of the eight breast cancer tissues examined relative to adjacent tissues in both immunohistochemistry (Figure 6A, top) and qRT-PCR analyses (Figure 6A, bottom). Conversely, expression of miR-205-5p was downregulated in all breast cancer tissue samples, as observed from *in situ* hybridization (Figure 6B, top) and qRT-PCR experiments (Figure 6B, bottom). Expression of miR-205-5p of lung cancer tissues was also similar to that of breast cancer tissues (Figure S4). MiRNAs circulate in the blood, which presents the advantage of being easily obtained from cancer patients. The relationship between Bcl-w and miR-205-5p expression was further verified using plasma from breast cancer patients treated with IR or showing metastasis to the lymph node. Bcl-w mRNA expression was higher in metastatic cancer plasma as well as breast cancer plasma, compared to normal plasma. Moreover, after radiotherapy, Bcl-w mRNA was dramatically increased in plasma of patients with metastatic cancer, compared to that of patients with non-metastatic cancer (Figure 6C). miR-205-5p expression was decreased in plasma of breast and metastatic cancer patients compared to normal patients. Notably, similar to data obtained with breast cancer patients subjected to radiotherapy, expression was attenuated significantly in plasma of metastatic cancer, compared to that of non-metastatic cancer patients (Figure 6D). Our data showed that expression of Bcl-w is negatively correlated with that of miR-205-5p in plasma of breast cancer patients (Figure 6E). In particular, Bcl-w expression was positively related to IR-induced aggressiveness and metastasis. These findings strongly support the feasibility of clinical application of Bcl-w and miR-205-5p expression analysis in blood of cancer patients.

### IR Hypermethylates miR-205-5p via Activating Src

**Figure 7:**
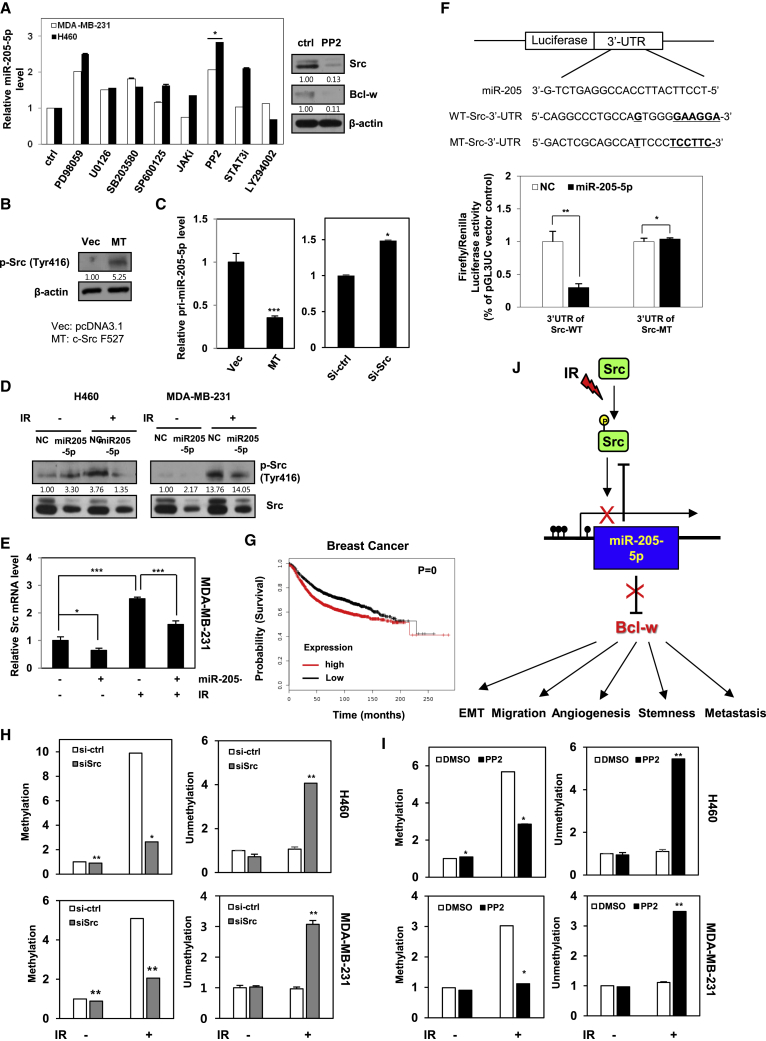
IR/Src/Bcl-w Signaling Axis Drives to Tumor Progression and Metastasis because of IR-Induced Hyper-methylation of miR-205-5p (A) Left, miR-205-5p expression levels were measured in H460 and MDA-MB231 cells, which were treated with inhibitors of ERK (PD98059), MEK (U0126), p38 (SB203580), JNK (SP600125), JAK, Src (PP2), STAT3, and PI3K (LY294002) by qRT-PCR. Right, expression levels of Src or Bcl-w protein by Src inhibitor (PP2) were determined by western blot analysis. β-actin was used as loading control. (B) H460 cells were transfected with vector control (pcDNA3.1) and Src-MT (c-SrcF527; active form). Expressions of Src phosphorylation (Tyr416) were shown by western blot analysis. (C) After transfection with Src-MT (left) or small interfering RNA Src (right) in MDA-MB-231 cells, the level of miR-205-5p was determined by qRT-PCR. (D and E) After H460 and MDA-MB-231 cells were exposed to IR (5 Gy) in presence or absence of miR-205-5p, levels of Src protein (D) and mRNA (E) were determined by western blot analysis and qRT-PCR, respectively. (F) Top, structure of reporter constructs containing Src 3′ UTR downstream of the luciferase open reading frame (ORF). pGL3UC-Src vectors containing the WT miR-205-5p binding site or a non-binding MT were constructed. Bottom, luciferase assays were performed with MDA-MB-231 cells, which were co-transfected with negative control, or miR-205-5p and pGL3UC-Src-WT, pGL3UC-Src mutant, or the empty vector for 48 h and were normalized by *pRL-CMV-Renilla*. (G) Kaplan-Meier analysis of the probability of survival as a function of relative expression of Src in breast adenocarcinoma tumors. (H and I) After H460 and MDA-MB-231 cells were exposed to IR (5 Gy) in presence or absence of siRNA (H) or inhibitor of Src (I), methylation or unmethylation of miR-205-5p CpG island was determined by qRT-PCR. (J) Scheme of IR-induced Src/miR-205-5p/Bcl-w signaling axis. IR induced downregulation of miR-205-5p expression through increasing its methylation by activating Src. As a result, IR-induced Src/miR-205-5p/Bcl-w axis is involved in tumor progression and metastasis of human cancer. Therefore, miR-205-5p may be useful as a genetic Bcl-w/Src-inhibiting therapeutic agent when treated with IR. All data are presented as the mean SD (*p < 0.05, **p < 0.005, ***p < 0.0005, Student’s t test).

To identify the key signaling molecules involved in the signaling mechanism underlying IR-mediated downregulation of miR-205-5p, H460 and MDA-MB-231 cells were treated with indicated inhibitors and levels of miR-205-5p expression were measured via qRT-PCR. The Src family kinase inhibitor, PP2, induced a dramatic increase in miR-205-5p expression in H460 and MDA-MB-231 cells (Figure 7A). To confirm these results, phosphorylation levels of Src were determined in H460 cells transfected with constitutively active forms (Src-MT; F527) of Src via western blot (Figure 7B). miR-205-5p expression was decreased in cells transfected with active Src-containing plasmid (Figure 7C, left) but increased in those with small interfering RNA (siRNA) against Src (Figure 7C, right), indicating downregulation of expression in association with Src phosphorylation in cancer cells. To further assess whether IR-regulated miR-205-5p involved in Src phosphorylation, phosphorylated Src protein and mRNA levels were determined after transfection with miR-205-5p mimic in H460 and MDA-MB-231 cells (either treated with IR or left untreated) via western blotting and qRT-PCR, respectively (Figures 7D and 7E). IR-exposed cells displayed enhanced phosphorylation and mRNA expression of Src, compared to negative control and miR-205-5p-transfected cells, further supporting IR-induced downregulation of miR-205-5p expression through Src phosphorylation. Conversely, miR-205-5p-transfected cells showed decreased Src expression. Src is a known target of miR-205-5p, identified based on the predicted miRNA target sites. We confirmed that Src is a target of miR-205-5p via the luciferase assay in MDA-MB-231 cells transfected with pGL3UC-Src vectors containing the WT miR-205-5p binding site or a non-binding mutant (Figure 7F). Consistently, low expression of Src was associated with increased probability of survival in breast adenocarcinoma patients, as estimated using Kaplan-Meier analysis^40^ (Figure 7G).

To confirm the relationship between miR-205-5p and Bcl-w or Src expression, MDA-MB-231 cells were transfected with siRNA of Bcl-w or Src in presence or absence of an inhibitor of miR-205-5p (anti-miR-205-5p). The miR-205-5p inhibitor promoted migratory potential (Figure S5A), invasiveness (Figure S5B), and sphere-formation ability (Figure S5C) as opposed to siRNA against Bcl-w or Src in MDA-MB-231 cells. In addition, when cells were co-treated with the miR-205-5p inhibitor and siRNA against Bcl-w or Src, tumorigenic phenotype and expression of its related components were decreased compared with only miR-205-5p inhibitor-transfected cells (Figures S5A–S5E). The results demonstrate that the miR-205-5p mimic acts as a tumor suppressor via suppressing Bcl-w and Src expression.

Epigenetic modification of miR-205 is reported to be induced in metastatic cancers.^43^, ^44^ To establish why biogenesis of miR-205-5p is decreased after IR exposure (Figure 4F), epigenetic modification of miR-205-5p by IR was examined. Hypermethylated miR-205-5p levels were augmented in IR-exposed cells, compared to siRNA- (Figure 7H, left) or chemical Src inhibitor-treated cells (Figure 7I, left). However, unmethylated miR-205-5p levels decreased in IR-exposed cells compared to siRNA- or chemical Src inhibitor-treated cells (Figures 7H and 7I, right). As shown Figure 7J, IR increased DNA methylation of the miR-205-5p promoter through Src phosphorylation, ultimately leading to decreased expression.

## Discussion

IR, in conjunction with chemotherapy and surgery, is effectively used as a major cancer therapy modality.^2^ However, surviving cancer cells acquire resistance after radiotherapy, following which the microenvironment of the tumor as well as distant tissues is altered, in turn, affecting multi-cellular responses, tissue remodeling, and metastasis.^1^, ^2^, ^3^, ^4^, ^5^, ^6^, ^7^ We investigated whether Bcl-w, identified as an oncogene in previous studies,^15^, ^16^, ^17^, ^22^ was induced by IR and involved in aggressive properties of cancer.

Bcl-w protein was upregulated in human lung and breast cancer tissues, relative to their normal counterparts (Figure 1A). Our results were consistent with previous reports of high expression of Bcl-w in several cancer types, including gastric, colorectal, cervical, breast, lung, and bladder cancer and glioblastoma multiforme, and its association with metastasis^15^, ^17^, ^22^ as a pro-oncogene.^23^ Notably, IR enhanced the expression of Bcl-w (Figure 1B), colony-forming ability, and living cell number (Figure S1) of human lung or breast cancer cells. Overexpression of oncogenes plays an important role in resistance to chemotherapy and radiotherapy.^45^ These results support the hypothesis that IR-induced Bcl-w is linked to therapeutic resistance of cancer cells and low mortality of patients through promoting cancer cell survival. Moreover, IR-induced Bcl-w accelerated pulmonary metastasis by inducing morphological changes consistent with epithelial-mesenchymal transition (EMT), migration, tube formation, and sphere-formation abilities, and expression of cancer stem-like cell markers and related factors in human lung and breast cancer cells (Figures 2 and 3). Our findings are consistent with previous studies showing an association of mesenchymal traits and cancer stem-like cell properties with resistance to chemotherapeutic drugs and radiation.^7^, ^17^, ^22^, ^46^ IR further promoted maintenance of stemness by regulating expression of cancer stem-like cell markers.^5^, ^6^ Based on these data, we propose that IR-induced Bcl-w is a critical resistance factor whose expression is stimulated by radiotherapy.

Since miRNAs are involved in cancer progression events, such as invasion and resistance to therapy,^47^ we focused on the possibility that IR may also regulate expression of miRNAs that contribute to malignant actions of cancer cells. miR-205-5p has been identified as an IR-downregulating and Bcl-w-targeting miRNA as well as a survival factor in patients using *in silico* miRNA target prediction software, such as Targetscan and miRanda (Figure 4A).^32^, ^33^ Expression patterns of Bcl-w and miR-205-5p were negatively correlated in both *in vitro* and *in vivo* systems, which were supported by clinical applicability, suggesting the possibility of using these molecules as therapeutic targets. miR-205-5p attenuated IR-induced tumor-progression events, such as migration, invasion, sphere formation, and expression of related factors, through suppression of Bcl-w in H460 and MDA-MB-231 cells (Figures 5A–5F). In addition, pulmonary metastasis was decreased in IR-treated mice in the presence of miR-205-5p, compared to IR-only-exposed mice (Figure 5G). Based on these data, we propose that miR-205-5p may be effectively applied as genetic therapy for reducing IR-induced resistance by suppressing malignant activities and metastasis. Bcl-w mRNA was upregulated significantly in plasma of metastatic patients after radiotherapy compared with in that of patients with non-metastatic cancer. While miR-205-5p expression was inhibited in plasma of breast cancer metastasis patients after radiotherapy compared with that of normal or breast cancer metastatic patients (Figures 6C and 6D). Consistently, expression of miR-205-5p was negatively correlated with that of Bcl-w in breast cancer patients (Figure 6E), supporting the feasibility of clinical application.

miR-205-5p expression was downregulated by Src phosphorylation in cancer cells. c-Src is widely expressed in many cancer tissues and plays an important role in the regulation of cell adhesion, growth, and differentiation.^48^ Src activation is additionally associated with pancreatic cancer progression and metastasis in mouse models.^49^ In our experiments, miR-205-5p inhibitor-induced tumorigenic phenotype and its related component expressions were rescued in cells transfected with siRNA against Bcl-w or Src (Figures S5A–S5E), suggesting that the miR-205-5p could act as a tumor suppressor by inhibiting Bcl-w and Src expression.

Previous studies have reported that DNA methylation contributes to the downregulation of miRNAs that act as tumor suppressors in cancer progression.^43^, ^44^ In this study, to determine the cause for decrease in biogenesis of miR-205-5p after IR exposure (Figure 4F), epigenetic modification of miR-205-5p expression by IR was examined. Our results are in keeping with previous studies showing that the CpG islands of miR-205-5p are hypermethylated in metastatic cancers.^43^, ^44^ IR enhanced hypermethylation of miR-205-5p through Src phosphorylation, leading to decreased miR-205-5p expression and, consequently, increased levels of Bcl-w that promoted malignant activity and metastasis. Based on the collective findings, we propose that miR-205-5p and Bcl-w, an important mediator of resistance in radiotherapy, can be potentially utilized as targets of concurrent genetic therapy to enhance the efficiency of radiotherapy in cancer patients.

## Materials and Methods

### Cell Culture

H460 (lung) and MDA-MB-231 (breast) were obtained from the Korea Cell Line Bank (KCLB). H460 and MDA-MB-231 cells were cultured in DMEM (Mediatech, Manassas, VA, USA) and RPMI 1640 media (Mediatech,), respectively. HUVECs were grown in endothelial cell growth medium MV2 with supplement mix (Promo Cell, Heidelberg, Germany). All medium was supplemented with 10% fetal bovine serum and 0.1% penicillin-streptomycin antibiotics (PAA Laboratories, Austria) in a humidified atmosphere of 5% CO_2._

### Antibodies and Inhibitors

Monoclonal antibodies against Bcl-w, Oct4, and Vimentin and polyclonal antibodies against phospho-Src (Tyr416) were purchased from Cell Signaling Technology (Beverly, MA, USA). Polyclonal antibodies against Slug, ZEB1, Sox2, Ang2, VEGF, and Ki-67 antibodies, monoclonal antibodies against MMP-9, c-Src, and β-actin were obtained from Santa Cruz Biotechnology (Dallas, TX, USA). Polyclonal Notch2 and Twist antibodies were purchased from Abcam. (Cambridge, MA, USA). The following pharmacological inhibitors were used in this study: inhibitors of phosphatidylinositol 3-kinase (PI3K) (LY294002), c-Jun N-terminal kinase (JNK) (SP600125), and Janus kinase (JAK) were purchased from Merck Millipore (Darmstadt, Germany). Inhibitors of p38 (SB203580), mitogen-activated protein kinase kinase (MEK) inhibitor (PD98059), and extracellular-signal-regulated-kinase (ERK) inhibitor (U0126) were obtained from Enzo Life Sciences (Lausen, Switzerland). Inhibitors of Src phosphorylation (PP2) and signal transducer and activator of transcription 3 (STAT3) were purchased from Calbiochem (San Diego, CA, USA). Inhibitor of DNMT1 (5-AzadC) were obtained from Abcam (Cambridge, MA, USA).

### Plasmid DNAs

pGL3UC vector constructs kindly were provided by V.N. Kim (School of Biological Sciences, Seoul National University, Korea).^50^ To perform the reporter construct, a DNA fragment of human Bcl-w 3′ UTR or Src containing putative miR-205-5p binding site were amplified by PCR and cloned into pGL3UC vector, respectively.^51^ The nucleotide sequences of primers for the amplification of the Bcl-w 3′ UTR were as follows: pGL3UC-Bclw-miR-205-5p WT, forward, 5′-AATCTAGAACACCAGAAACTCAGAGC-3′, reverse, 5′-AAGAATTCGGTCTAGTCAGTGGTTT T-3′; pGL3UC-Bclw-miR-205-5p MT, forward, 5′-GTCCTTGGGGAGATG**CCTT**GG GTGGGGAGCTGAG-3′, reverse, 5′-CTCAGCTCCCCACCC**AAGG**CATCTCCCCAAG GAC-3′;^43^ pGL3UC-Src-miR-205-5p WT, forward, 5′-AAT CTA GAC CAT GTG CGT CCA TAT TT-3′, reverse, 5′-AAG AAT TCA CAA AGG GCC TTA GGC AG-3′; pGL3UC-Src-miR-205-5p MT, forward, 5′-CTGCCAGTGGG**TCCTTC**GGCCAAGCAGTGCCTGCCTA**ATCCCA**TTCAAC TTTTC-3′, reverse, 5′-GAAAAGTTGA**ATGGGAT**TAGGCAGGCACTGCTTGGCC**GAAGGA**CCC ACTGGCAG-3′.^51^ The active form (F527) of c-Src was provided by S.J. Lee (Department of Life Science, Hanyang University, Korea). pcDNA3.1 vector only was also transfected as a control.

### RNA Oligoribonucleotides and Transfection

Synthetic miRNA mimics or inhibitors were synthesized by IDT incorporation (Integrated DNA Technologies, Iowa, USA), as RNA duplexes designed from the sequences of miR-205-5p (5′-UCCUUCAUUCCACC-3′) using 5′-UGAAUUAGAUGGCGAUGUUTT-3′ for the negative control. The inhibitor of miR-205-5p was a 2′-O-methyl-modified oligoribonucleotide single strand with the sequence as 5′-CAGACUCCGGUGGAAUGA AGG-3′. All siRNAs (Bcl-w and Src) and shRNA (sh-Bcl-w) were purchased from Santa Cruz Biotechnology. siRNA duplexes, shRNA, miRNAs, and plasmids were introduced into cells using Lipofectamine 2000 reagent (Thermo Fisher Scientific, Waltham, MA, USA) according to the manufacturer’s instructions.

### Irradiation

Cancer cells were exposed to γ-rays with 137Cs γ-rays source (Atomic Energy of Canada, Canada) with a dose rate of 3.81 Gy/min.

### Western Blot Analysis

Cells were subjected in RIPA buffer to protease and phosphatase inhibitor cocktail tablet (Roche, Indianapolis, IN, USA). Total protein extract was separated by SDS-PAGE, electro-transferred to the polyvinylidene fluoride (PVDF) membrane (Millipore Corporation, MA, USA), and blocked in 5% skim milk in Tris-buffered saline Tween 20 (TBST) (10 mM TrisHCl [pH 8.0], 150 mM NaCl, and 0.05% Tween 20). The indicated primary antibody was reacted as 1:1,000∼5,000 overnight at 4°C. The secondary antibody, mouse, rabbit, and goat were reacted as 1:5,000∼10,000 for 1 h at room temperature and detected by chemiluminescence with an enhanced chemiluminiscence system (WesternBright ECL, Advansta, CA, USA). Bands of western blotting were quantified using the ImageJ program (NIH, USA).

### Wound-Healing Assay

Cells were seeded on 6-well culture plates and scratched with a plastic tip for mimic of wound injury. After 16–24 h in an incubator, migrating cells were assessed by the closure of the wound area, as described previously.^13^

### Transwell Invasion Assay

Invasion assay was performed using Matrigel-transwell chambers (8 μm pore). Invasion assay was performed using Matrigel (BD Biosciences, San Jose, CA, USA)-coated transwell chambers (Corning, Corning, NY, USA). Cells (2.5 × 10^4^ cells/well) were placed in the upper transwell chamber, and medium containing 0.1% BSA was added to the lower chamber. The next steps were done according to the manufacturer’s instructions. After incubation for 16 h, the cells that migrated to the lower surface of the filter were fixed and stained using Diff-Quick kit (Fisher Scientific, PA, USA). The stained cells were counted under a light microscope (Mitoti AE31 series, Trinocular Inverted MIC).

### Tube Formation Assay

HUVECs were seed on 96-well plates coated with Matrigel (BD Biosciences) as described.^15^ HUVECs were transfected with small interfering RNA against Bcl-w and incubated in serum-free medium for 24 h. Total tube numbers were counted and compared from three different fields using inverted microscope.

### Sphere-Formation Assay

Sphere-formation assay was performed as described previously.^22^ The indicated cells (1 × 10^5^) were resuspended in DMEM-F12 (Cellgro, Manassas, VA, USA) containing B27 (1:50) (Gibco, USA) as cancer stem cell permissive medium and grown for 5–10 days. Spheres were counted with a diameter > 20 μm under an inverted microscope (Miotic AE31 series).

### Three-Dimensional Culture

24-well plate pre-coated with 60 μL of Matrigel (BD Biosciences) was prepared according to manufacturer’s instructions. 50–100 cells of H460 and MDA-MB-231 cells suspended in 600 μL of growth medium with Matrigel were seeded on the top of the Matrigel layers. After incubation in 37°C to form colonies, colonies were photographed every 5 days.

### Luciferase Dual-Reporter Assay

Luciferase activity was measured using dual-luciferase reporter assay system (Promega, Madison, WI, USA) according to the manufacturer’s instructions and normalized to *Renilla* luciferase activity. Cells reached around 50% confluency in 24-well culture plates and were then co-transfected for 48 h with reporter plasmid (200 ng), pRL-CMV-*Renilla* (Promega) plasmid (1 ng), and miRNA using Lipofectamine 2000 (Thermo Fisher Scientific). Luciferase activity was measured using dual-luciferase reporter assay system (Promega) according to the manufacturer’s instructions and normalized to *Renilla* luciferase activity. All experiments were performed in triplicate.

### Clonogenic Assay

Cells were seeded into 6-well plates at a density of 100–10,000 cells/well and were incubated overnight. After exposure with IR (0, 2, 4, 6, or 8 Gy), the cells were cultured for 14 days. Colonies were stained with crystal violet staining solution and counted. The experiments were performed in triplicate.

### Real-Time qRT-PCR

The first-strand cDNA was synthesized by reverse transcription and was amplified using an RCR cycler (Bio-Rad, CA, USA). Total RNA was extracted using the TRIzol reagent (Favorgen Biotech., Taiwan) following the manufacturer’s protocol. RNA was used for the reverse transcription reaction using the Tetro cDNA Synthesis Kit (Bioline, London, UK). Real-time PCR was performed using Mir-X miRNA qRT-PCR SYBR kit (Clontech Laboratories, Mountain View, CA) according to manufacturer’s protocol. Primer sequences are as follows: 5′-CATCAAGGGCATTCAGGAGC-3′ (forward) and 5′-AGAACACAGCCTTCTCCTCC-3′ (reverse) for MMP-2; 5′-TCGTGGTTCCAACTCGGTTT-3′ (forward) and 5′-GGTTTCCCATCAGCATTG CC-3′ (reverse) for MMP-9; 5′-GAG AATTCTTTGGAACTCGCAGTCCTCT-3′ (forward) and 5′-TCTCTTTCCACCCAGGT GTC-3′ (reverse) for Bcl-w; 5′-CAT CTCTGCCCCCTCTGCTGA-3′ (forward) and 5′-GGA TGACCTTGCCCACAGCCT-3′ (reverse) for glyceraldehyde 3-phosphate dehydrogenase (GAPDH). Expression of mRNA was calculated using the 2–ΔΔCt method and normalized with GAPDH: 5′-TCCTTCATTCCACC GGAGTCTG-3′ (forward) for mature miR-205-5p; 5′-TGGCCAGGTAATGCAGTGT-3′ (forward) for primary miR-205-5p; 5′-TCCTTCATTCCACCGGAGTCTG-3′ (forward) for precursor miR-205-5p; 5′-GAACTTCACTCCACTGAAATCTG-3′ (reverse) for primary and precursor miR-205-5p. The cycle threshold (Ct) values are similar to within 0.5 among triplicates. The primer was designed for miR-205-5p and U6, which yielded a 2−ΔΔCt value and was used for normalization. The experiments were performed in triplicate.

### MSP

Genomic DNA was converted with the EZ DNA methylation-gold kit from Zymo Research (Irvine, CA, USA). After amplification of the bisulfite-converted DNA with specific primers for the miR-205-5p, DNA methylation levels were analyzed by qRT-PCR and agarose gel running as previously described.^43^ Primer sequences are as follows: 5′-GAGTTTAAGTTGCGTATGGAAGC-3′ (forward) and 5′-AAAACAAATATT TCTTTTATAATCCGA A-3′ (reverse) for methylated miR-205-5p; 5′-GGAGTTTAAGTTGTGTATGGAAGTG-3′ (forward) and 5′-AAAACAAATATTTCTT TTATAA TCC AAA-3′ (reverse) for unmethylated miR-205-5p.

### Animal Experiments

Six-week-old BALB/c female nude mice were obtained from Orient Bio (Seongnam, Korea). Highly metastatic MDA-MB-231 cells as human breast cancer cells were kindly provided by S.J. Lee (Department of Life Sciences, Hanyang University, Korea). Metastatic MDA-MB-231 cells with sh-control or sh-Bcl-w (1 × 10^6^ cells) were injected into the mammary fat pad of the mice. At a tumor volume of 200–250 mm^3^, tumors were irradiated using a customized shielding device with 2.5 Gy per day for 3 days.^39^ Tumor sizes were measured with a caliper (calculated volume = 0.5 × long diameter × short diameter)^2^ as described.^52^ Metastatic pulmonary nodules in the lungs were counted. The primary tumors and pulmonary tissues were formalin fixed and paraffin embedded for immunohistochemistry (IHC) or homogenized to prepare the tissue lysates for western blot analysis.^53^ This study was reviewed and approved by the Institutional Animal Care and Use Committee (IACUC) of Korea Institute of Radiological & Medical Science.

### Clinical Samples

Human specimens were provided from the Radiation Tissue Resources Bank of Korea Cancer Center Hospital and Korea Institute of Radiological and Medical Sciences (KIRAMS) Radiation Biobank (KRB). All samples used in this experiment have completed Institutional Review Board (IRB) approval (K-1608-002-048) in the Korea Institute of Radiological and Medical Sciences (KIRAMS).

### IHC

Paraffin-embedded tumor tissues were sectioned, deparaffinized, and stained with H&E as previously described.^22^ Using paraffin-embedded tumor tissues, detection of the antibody signal was performed with the LSAB2 System-HRP (K0672; Dako) and the liquid DAB+ Substrate Chromogen System (K3468; Dako) according to the manufacturer’s instructions. Immuno-positive cells and staining intensities were quantified with a cellSens (Olympus, Tokyo, Japan).

### *In Situ* Hybridization

The rehydrated tissue sections were transferred into a 3% H_2_O_2_ and protease buffer for inactivation of endogenous peroxidase. The sections were treated with pepsin solution diluted in 3% citric acid and were hybridized overnight at 59°C with a 50-nM digoxigenin (DIG)-labeled locked nucleic acid (LNA)-based probe specific for miR-205-5p or LNA-src-miR negative control probe (Exiqon, Denmark). After rinsing in the washing buffer, the sections were blocked in blocking buffer. A specific antibody, DIG-AP (Fab fragments), was applied to the sections.

### Kaplan-Meier Curves

Kaplan-Meier analyses of survival in breast cancer were performed by the open source KM Plotter (http://www.kmplot.com).^40^

### Statistical Analysis

The Spearman correlation was calculated for verify the relationships Bcl-w and miR-205-5p level using Graphpad Prism (version 5.0, GraphPad Software). All data are presented as mean ± SD. Statistical calculations were performed with Student’s t test. Differences were considered significant at p < 0.05.

## Author Contributions

I.H.B. supervised the work; E.S.K., J.Y.C, and S.J.H. performed research and analyzed data; I.H.B. and E.S.K. designed the experiments and drafted the manuscript. All authors discussed the results and commented on the manuscript.

## Conflicts of Interest

The authors declare no potential conflicts of interest.

## Supplemental Information

Document S1. Figures S1–S5

Document S2. Article plus Supplemental Information
