# Supplementary material for: Hypermethylation of miR-205-5p by IR Governs Aggressiveness and Metastasis via Regulating Bcl-w and Src
Source: Mol Ther Nucleic Acids. 2018 Dec 31;14:450–64. doi: 10.1016/j.omtn.2018.12.013 (PMC6369268; doi:10.1016/j.omtn.2018.12.013)
Supplement: Document S1. Figures S1–S5 [file mmc1.pdf]

OMTN, Volume *14*

Supplemental Information

Hypermethylation of miR-205-5p by IR Governs Aggressiveness and Metastasis via Regulating Bcl-w and Src

Eun Sook Kim, Jae Yeon Choi, Su Jin Hwang, and In Hwa Bae

Figure S1

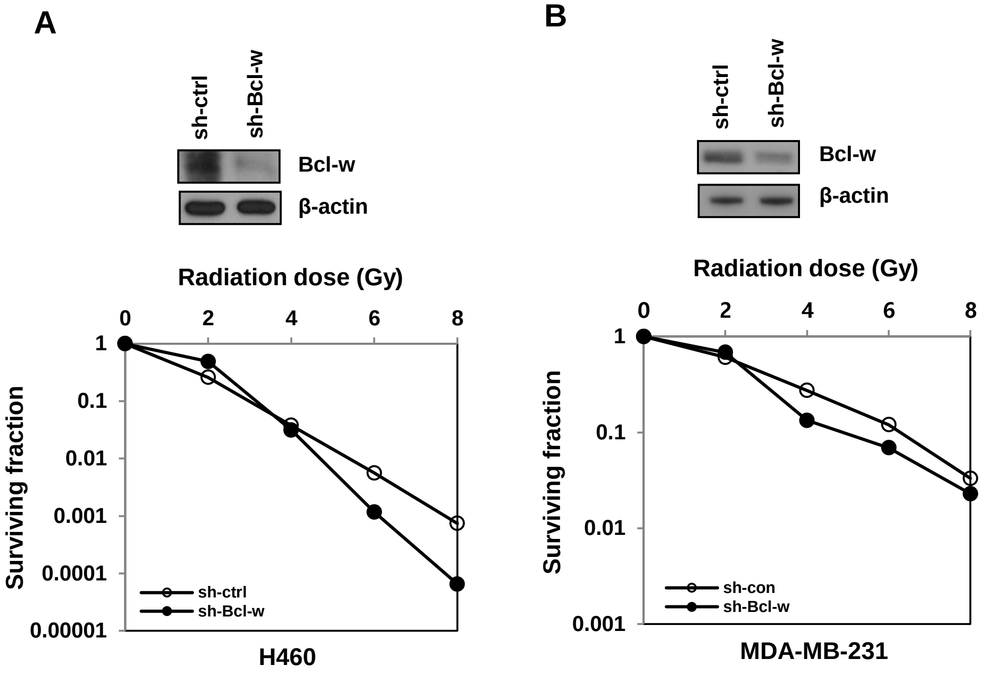

Supplementary Figure S1. IR-induced Bcl-w is related to resistance of cancer cells. After treatment of IR (2, 4, 6, and 8 Gy), clonogenic assay was conducted in sh-Bcl-w or sh-control-transfected cells, H460 (A) and MDA-MB-231 (B) for 14 days. Colonies of surviving cells were counted and expressed as a natural log was plotted.

Figure S2

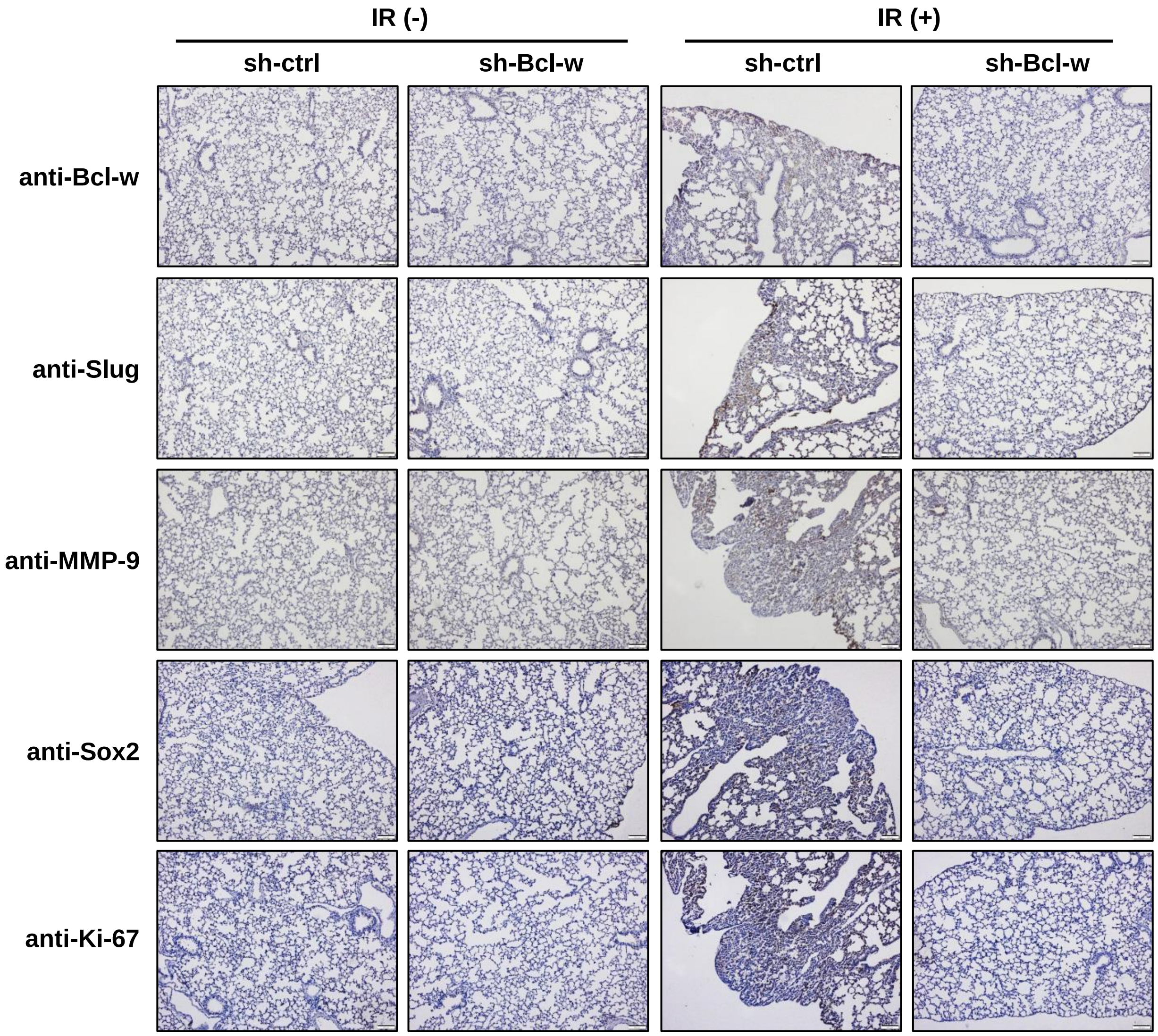

Supplementary Figure S2. IR induces Bcl-w-related signaling components. Expressions of Bcl-w, mesenchymal traits (Slug), invasiveness (MMP-9), stemness maintenance (Sox2), and proliferation marker (Ki-67) were showed in metastatic pulmonary tissues by immunohistochemistry staining (Scale bar, 100 ㎛).

Figure S3

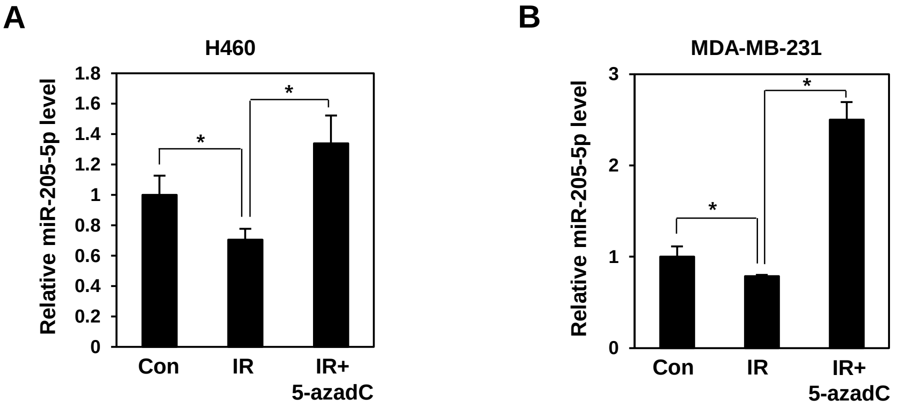

Supplementary Figure S3. IR downregulates miR-205-5p expression via its methylation. After IR exposed, H460 (A) and MDA-MB-231 (B) cells were treated with 5uM 5-Aza-2'-deoxycytidine (5-AzadC, DNMT1 inhibitor) for 48h. Expression of miR-205-5p was measured by qRT-PCR.

Figure S4

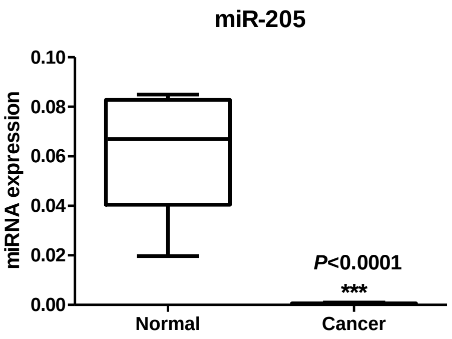

Supplementary Figure S4. Expression of miR-205-5p is suppressed in lung cancer tissue of patients. Analysis of miR-205-5p expression in lung cancer tissues compared to adjacent tissues by qRT-PCR.

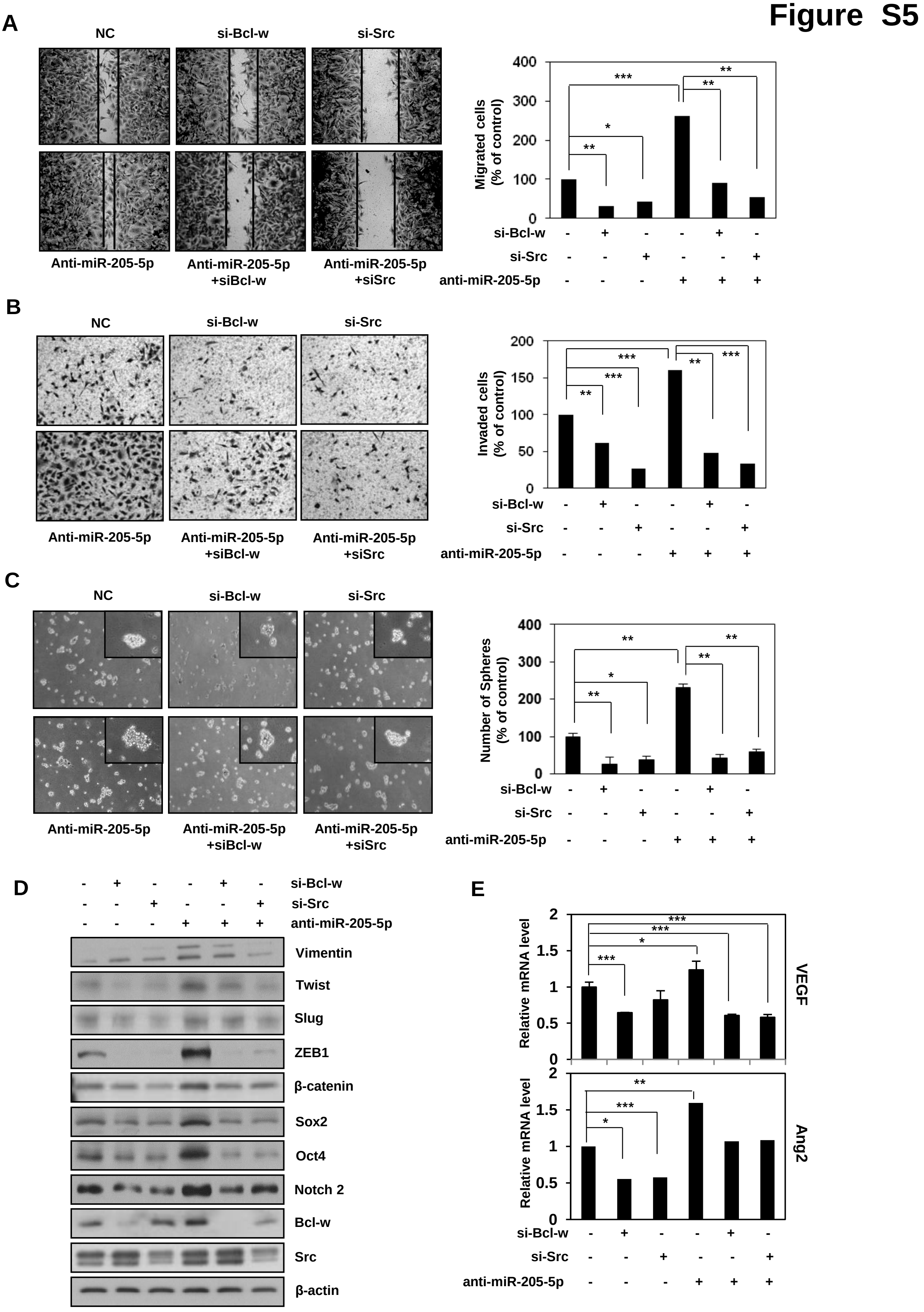

Supplementary Figure S5. miR-205-5p mimic inhibits IR-induced Src-Bcl-w signaling axis which induced migration, invasion, and stemness. Migration (A), invasion (B), sphere-formation assays (C), western blot analysis (D), and qRT-PCR (E) were performed using siRNA against Bcl-w and Src, and inhibitor of miR-205-5p in MDA-MB231 cells. All data are presented as the mean S.D. (* $P < 0.05$, ** $P < 0.005$, *** $P < 0.0005$, Student's t-test).
